# ParaMed: a parallel corpus for English–Chinese translation in the biomedical domain

**DOI:** 10.1186/s12911-021-01621-8

**Published:** 2021-09-06

**Authors:** Boxiang Liu, Liang Huang

**Affiliations:** 1 Institute of Deep Learning, Baidu Research, Sunnyvale, USA; 2 School of Electrical Engineering and Computer Science, Oregon State University, Corvallis, USA

**Keywords:** Machine translation, Natural language processing, Text mining

## Abstract

**Background:**

Biomedical language translation requires multi-lingual fluency as well as relevant domain knowledge. Such requirements make it challenging to train qualified translators and costly to generate high-quality translations. Machine translation represents an effective alternative, but accurate machine translation requires large amounts of in-domain data. While such datasets are abundant in general domains, they are less accessible in the biomedical domain. Chinese and English are two of the most widely spoken languages, yet to our knowledge, a parallel corpus does not exist for this language pair in the biomedical domain.

**Description:**

We developed an effective pipeline to acquire and process an English-Chinese parallel corpus from the New England Journal of Medicine (NEJM). This corpus consists of about 100,000 sentence pairs and 3,000,000 tokens on each side. We showed that training on out-of-domain data and fine-tuning with as few as 4000 NEJM sentence pairs improve translation quality by 25.3 (13.4) BLEU for en$\rightarrow$zh (zh$\rightarrow$en) directions. Translation quality continues to improve at a slower pace on larger in-domain data subsets, with a total increase of 33.0 (24.3) BLEU for en$\rightarrow$zh (zh$\rightarrow$en) directions on the full dataset.

**Conclusions:**

The code and data are available at https://github.com/boxiangliu/ParaMed.

## Background

Biomedical translation is used across various life science disciplines. Example applications include translation of clinical trial consent forms, regulatory documents, and interpretation within point-of-care facilities [1, 2]. Biomedical translation requires up-to-date domain knowledge and fluency in the source and target languages. Such requirements make it challenging to train qualified translators and costly to generate high-quality translations.

Recent advances in machine translation have demonstrated translation quality arguably on par with professional human translators in select domains [3]. Supervised training of machine translation models usually benefits from large amounts of parallel corpora and such effect is the most evident for neural machine translation models. However, the collection and alignment of parallel corpora requires significant time and labor, and such datasets are not available for all domains or language pairs.

Machine translation in the biomedical domain is characterized by a long tail of medical terminology. For example, the Unified Medical Language System (UMLS) developed by the National Institute of Health contains over 2 million names for over 900,000 concepts [4], much larger than the set of common English words. Therefore, domain adaptation (training on out-of-domain data and testing on in-domain data) from the general domain to the biomedical domain is challenging.

Two prevailing challenges impact biomedical translation quality when training is done on general-domain data. Biomedical concepts unseen in the general-domain training set (covariate shift) are difficult to translate accurately. Most medical terminologies, such as the word “oncogenesis”, falls into this category. Additionally, concepts that appear in both biomedical domain and general domain but with different semantics present a second challenge. For example, “*primary* care” is translated to Chinese as “  ” whereas “*primary* element” is translated as “  ”.

Various domain adaptation techniques have been developed. Synthetic data generation such as forward and backward translation [5] aims to augment out-of-domain parallel data with monolingual in-domain data. Data selection methods aim to select in-domain examples from general domain data [6]. Fine-tuning with a small amount of in-domain data has been shown to substantially improve translation quality [7].

While the need for biomedical parallel corpora is evident, they are not available for all language pairs. In a literature survey, we found that existing public biomedical parallel corpora are between European Languages (Table 1). The UFAL Medical Corpus covers language pairs from English to Czech, German, Spanish, French, Hungarian, Polish, Romanian and Swedish. The ReBEC dataset [8] contains Portuguese and English parallel texts obtained from 1,188 clinical trial documents in the Brazilian Clinical Trials Registry. The 2020 Conference on Machine Translation (WMT20) Biomedical Translation Workshop [9] provides training sentence pairs from Medline abstracts between English and Spanish/German/Portuguese/French/Italian/Russian, but only test sentence pairs for English and Chinese. The Khresmoi dataset [10] samples 1,500 English sentences from medical documents. These sentences are manually translated into Czech, French, German, Hungarian, Polish, Spanish, and Swedish. The MeSpEn dataset [11] contains English and Spanish parallel text collected from IBECS (Spanish Bibliographical Index in Health Sciences), SciELO (Scientific Electronic Library Online), Pubmed and MedlinePlus. Furthermore, we found that existing public English-Chinese parallel corpora are outside of the biomedical domain. The OPUS corpora contain English-Chinese translation from numerous sources such news, speeches, and movie subtitles [12]. Perhaps the most closely related is the UM-corpus. It contains parallel text from eight different domains, one of which is science and technology [13].

**Table 1:** Existing parallel corpus in the biomedical domain contains only European languages

Corpus	Language Components
UFAL	cs, de, en, es, fr, hu, pl, ro, sv
ReBEC	en, pt
WMT19	de, en, es, fr, pt
Khresmoi	cs, de, en, es, fr, hu, pl, sv
MeSpEn	en, es

cs: Czech, de: German, en: English, es: Spanish, fr: French, hu: Hungarian, pl: Polish, ro: Romanian, sv: Swedish

The New England Journal of Medicine (NEJM) provides Chinese translations of its publications dating back to 2011 (http://nejmqianyan.cn/). The website repository currently hosts nearly 2,000 articles, with new articles added weekly. These articles include original research articles, clinical case reports, review articles, commentaries, Journal Watch (viz. article highlights), etc. The articles are translated by professional translators and proofread by members of the NEJM editorial team. For research articles, translations on statistical analysis are proofread by statisticians who are native Chinese speakers.

**Fig. 1:**
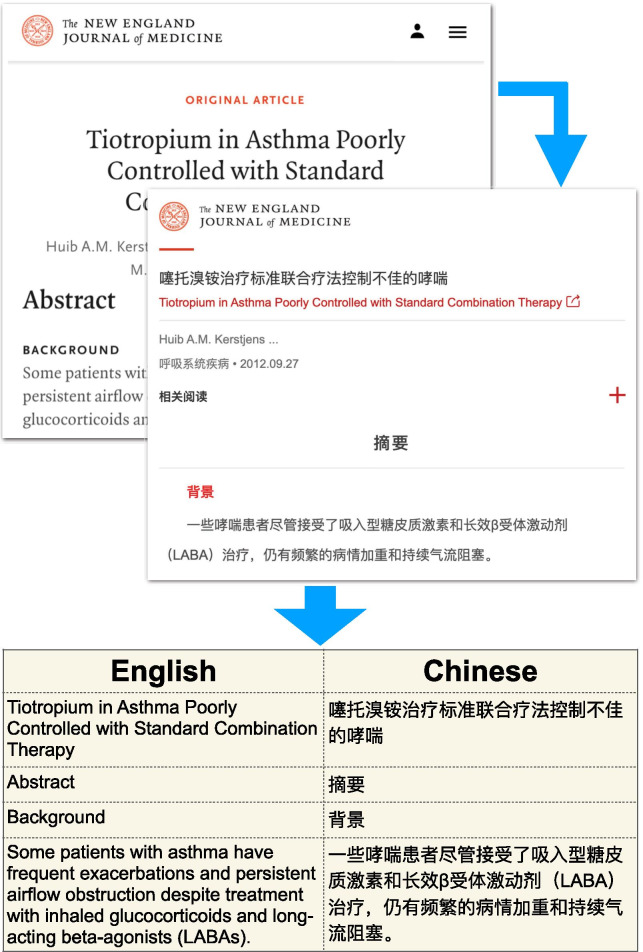
An overview of the ParaMed corpus construction. The input is the NEJM website and the output is a Chinese/English parallel corpus

In this study, we present an English–Chinese parallel corpus in the biomedical domain constructed from NEJM (Fig. 1). We provide sentence-aligned bitext for 1966 article pairs, totaling 97,441 sentence pairs. Further, we show that training a baseline model with the 2018 Conference on Machine Translation (WMT18) newswire data [14] and fine-tuning the model with the ParaMed dataset will significantly improve translation quality over the baseline model, suggesting that the ParaMed dataset will be useful in improving biomedical translation quality.

Our contributions are the following:

- We present the first English-Chinese parallel corpus in the biomedical domain. We only use the *open-access* portion of NEJM articles to comply with their editorial policy.
- We provide an end-to-end pipeline for constructing parallel corpus using web-crawled text. We compare several software packages for sentence boundary detection and alignment and provide guidelines on their performance in the biomedical domain.
- We show that fine-tuning on as few as 4,000 sentence pairs from ParaMed can improve translation quality by 25.3 (13.4) BLEU for en$\rightarrow$zh (zh$\rightarrow$en). Translation quality continues to improve at a slower pace on larger datasets, finishing at an increase of 33.0 (24.3) BLEU for en$\rightarrow$zh (zh$\rightarrow$en) on the full dataset.

## Construction and content

### Standard approaches to parallel corpus construction

Construction of a sentence-aligned parallel corpus from multilingual websites involves the following steps.

1. Documents in desired languages are crawled from multi-lingual websites.
2. Plain texts are extracted from crawled documents and normalized to remove special characters.
3. Documents from both languages are matched according to their contents.
4. Within each document, paragraphs are broken down into individual sentences.
5. Sentences are subsequently aligned into sentence pairs.
6. Aligned sentence pairs are filtered to remove duplicated and low-quality pairs.

While the first two steps are well-established engineering tasks, the last four are under active research. For step 3, the 2016 Conference on Machine Translation (WMT16) hosted a shared task for bilingual document alignment [15], in which the best entry relied on matching distinct bilingual phrase pairs [16]. For step 4, Read *et al.* [17] systematically evaluated nine existing tools for sentence boundary detection, among which LingPipe [18] and Punkt [19] are the top performers in the biomedical domain. Sentence alignment (step 5) is arguably the most challenging step. Compared with document alignment, sentence alignment uses a smaller amount of text but has more permutations. Various methods have been proposed, among which are length-based algorithm [20], lexicon-based algorithm [21–23], and translation-based algorithm [24, 25], with no consensus on the best performer. For step 6, WMT18 hosted a shared task on parallel corpora filtering [26], in which the best performer used dual conditional cross-entropy filtering [27].

**Fig. 2:**
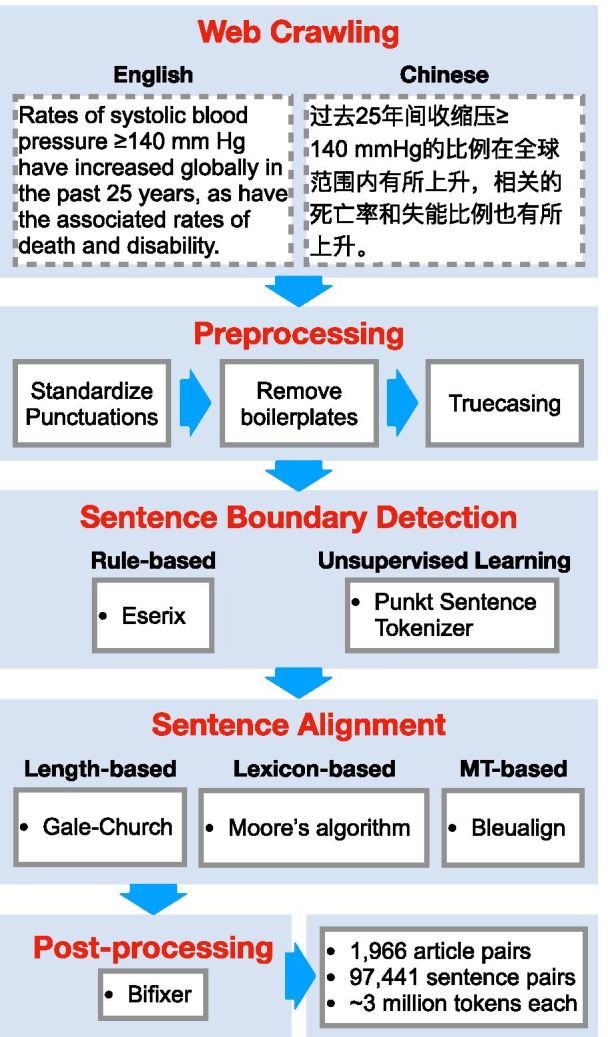
The overall pipeline to construct the ParaMed dataset. NEJM webpages were crawled using Selenium. Various preprocessing steps were carried out to standardize punctuations and remove boilerplate texts. We tested two methods for splitting paragraphs into sentences, and three methods to align English and Chinese sentence into translated pairs. Duplicated sentence pairs were removed at the end

The NEJM website provides hyperlinks between Chinese and English article pairs, allowing us to skip document alignment (step 3). Otherwise, we followed the best practices outlined therein and adapt them to our project (Fig. 2).

### The New England Journal of Medicine Dataset

The Chinese website of the New England Journal of Medicine (https://www.nejmqianyan.cn/) provides open-access Chinese translations dating back to 2011. All articles were translated sentence for sentence by professional translators, with occasional sentence concatenation and division for fluency. In other words, one English sentence can be split into two or more Chinese sentences and vice versa. Translations were proofread by members of the editorial team and research articles were additionally proofread by statisticians. The Chinese translations are organized chronologically, making the content easy to crawl. Correspondent article pairs are connected via hyperlinks, eliminating the need for document alignment.

### Web crawling

We used Selenium [28] to crawl all available Chinese and English articles. While paragraph orderings are maintained across languages, locations of display items—figures, tables, and associated captions—are shuffled. We removed display items to keep content orders identical across English and Chinese. The English NEJM website contains untranslated auxillary contents such as job boards and visual advertisements. We instructed Selenium to ignore auxillary contents as these interjections make sentence alignment challenging. Chinese NEJM translations are cleaner but contain boilerplate sentences such as names of translators. These boilerplate contents were removed during preprocessing.

### Preprocessing

We truecased letters and standardized punctuations for crawled articles with moses [29], and subsequently performed stitching and filtering described below.

#### Stitching incorrectly split sentences

A single sentence is occasionally split incorrectly due to inappropriate HTML tags. In Chinese articles, we found that sentence breaks can be inserted by mistake before citations and before punctuations. To stitch them, we assigned any text segment consisted only of citations and/or punctuations to its preceding sentence. For English, we noticed that the phrase “open in new tab” always incorrectly break a full sentence into two halves. We concatenated flanking sentences and remove the said phrase.

#### Filtering

Because display items and references are untranslated, we filtered out the following content for both languages:

- Figures and figure captions
- Tables and table legends
- Reference sections

Further, we removed content specific for either language. For Chinese, we removed any information about translators. For English, we removed:

- Video
- Interactive graphic
- Audio interview
- Visual abstract
- Quick take (video summary)

### Sentence boundary detection (SBD)

Chinese sentences are concluded by three full-stop punctuations {  }. These punctuations are used exclusively for sentence separation. Unlike European languages, they do not double as decimal points or other linguistic markers. Further, Chinese quotation marks appear before sentence breaks, making it easy to detect sentence boundaries. Breaking English sentences is more challenging due to punctuation overloading.

Read et al. [17] showed that punkt, an unsupervised sentence tokenizer, is a top performer on biomedical corpora. We trained punkt on our ParaMed corpus and used the learned parameters to break sentences. Since punkt does not support the Chinese language, we implemented a custom regex-based tokenizer to split Chinese paragraphs into sentences.

Further, we tested a rule-based system eserix [30] designed to process the United Nations parallel corpus and has built-in support for both Chinese and English [31]. However, the default rules do not include commonly used abbreviation in biomedical literature, such as the word “Vol.” as an abbreviation for “Volume”. We added rules into the eserix ruleset specifically for the ParaMed corpus.

### Sentence alignment

While many methods have been proposed for sentence alignment, there is no consensus on their performance in the biomedical domain. We focused on three main classes of methods: length-based, lexicon-based, and translation-based methods. We drew one method from each class: Gale-Church (length-based), Microsoft Aligner (lexicon-based), and Bleualign (translation-based). The Gale-Church algorithm finds sentence pairs based on the assumption that the lengths of source and target sentences should be similar [20]. The Microsoft Aligner integrates word correspondence with sentence lengths to search for sentence pairs [21]. Bleualign compares original and translated texts to search for anchor sentences and subsequently aligns the rest with the Gale-Church algorithm [25]. To compare these methods, we established a test set by manually aligning 1,019 sentence pairs from 12 articles. Table 2 shows the distribution of alignment types. Nearly 95% of all alignments are one-to-one. An example of one-to-many alignment is shown in Table 3.

**Table 2:** Alignment counts in manually aligned sentence pairs, in which the majority are 1–1 alignments

zh-en	Count	Percent
0–1	10	1.0%
1–0	11	1.1%
1–1	964	94.6%
1–2	17	1.7%
2–1	15	1.5%
2–2	1	0.1%
2–3	1	0.1%

**Table 3:**
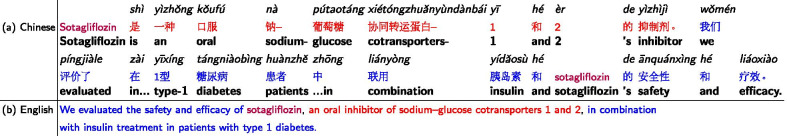
An example 1-to-2 alignment for clause breaking. The red text denotes the English clause corresponding to the first Chinese sentence. Sotagliflozin is cited once in the English sentence, but repeated in two Chinese sentences

### Post-processing

Medical literature is highly structured. Certain sections such as the abstract, introduction, methods, results and discussion are almost universal across articles. We removed duplicated header and other repeated text with bifixer [32].

### Training, development and test split

We selected 2102 sentence pairs from 39 latest articles as the test set and 2036 sentence pairs from the next latest 40 articles as the development set. The remaining 93,303 sentence pairs constitute the training set. To avoid data leakage, all sentences from each articles must be in one of either train, development, and test set.

### Model architecture

We used the transformer model [33] in OpenNMT with 6 layers, each with an output size of 512 hidden units [34]. We used 8 attention heads and sinusoidal positional embedding. The final hidden feed-forward layer is of size 2,048. In addition, we used an LSTM [35] in OpenNMT with 512 hidden units.

### Hardware and training procedure

We trained baseline transformer and LSTM models on the English-Chinese parallel corpus from WMT18 [36] consisting about 24.8 million sentence pairs. Sentences are encoded with Byte-Pair Encoding [37] with vocabularies of 16,000 tokens for each language. Sentence lengths are capped at 999 tokens, enough to accommodate most sentences. We trained these models on 8 Nvidia TitanX GPUs. For the transformer model, we used the Adam optimizer [38] with $lr = 2$, $\beta _1 = 0.9$, $\beta _2 = 0.997$ and 10,000 warm-up steps. We applied dropout with $p_{d} = 0.1$ and label smoothing with $\epsilon _{ls} = 0.1$. The model was trained for 500,000 steps in total. The training procedure took 4.5 days. We fine-tuned the baseline model on ParaMed for 100,000 steps with identical parameters. To establish a second comparison, we trained a transformer model *de novo* on the ParaMed corpus. For the LSTM model, we used the Adam optimizer with $lr = 0.001$, $\beta _1 = 0.9$, $\beta _2 = 0.999$, and label smoothing with $\epsilon _{ls} = 0.1$.

## Utility and discussion

### Statistics on crawled articles

**Fig. 3:**
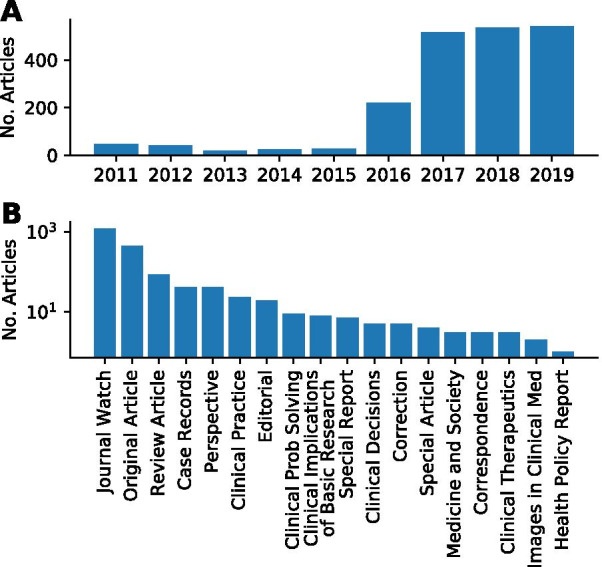
Distribution of articles by year and by article type. **A** The number of translated articles has been on the rise since the year 2015. **B** Journal Watch, original articles, and review articles are the top three article types

The earliest official translation by NEJM dates back to 2011, and the number of translated articles has been on the rise year over year. Journal Watch (article highlights) leads in the number of articles, followed by original research and review articles. Figure 3 shows the distribution of articles by year and type.

### Comparing pre- and post-filtered corpora

**Fig. 4:**
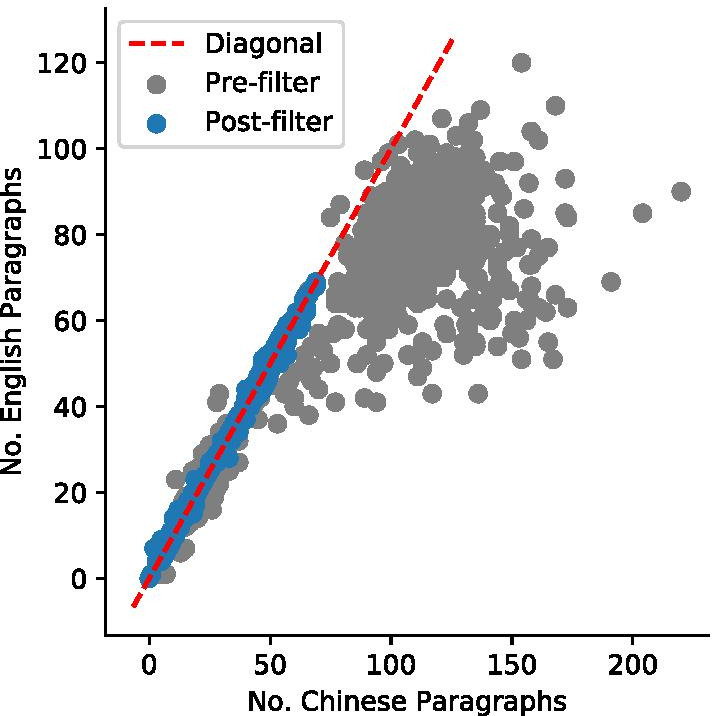
Number of Chinese and English paragraphs are closer post-filtering compared to pre-filtering. Each point represents an English/Chinese article pair

To remove untranslated text and display items, we manually compared the corresponding Chinese and English articles, identified HTML divisions to be filtered, and implemented a rule-based system to automatically filtered out matching HTML divisions (“Filtering” section). Figure 4 compares the number of Chinese and English paragraphs in each article pair before and after filtering. Before filtering, the number of Chinese paragraphs exceeds that of English for numerous articles, indicated by the grey sub-diagonal cloud. This is due to the various untranslated and boilerplate texts within the articles. The number of English and Chinese paragraphs in each article become closer after filtering.

### Comparing sentence boundary detection algorithms

**Fig. 5:**
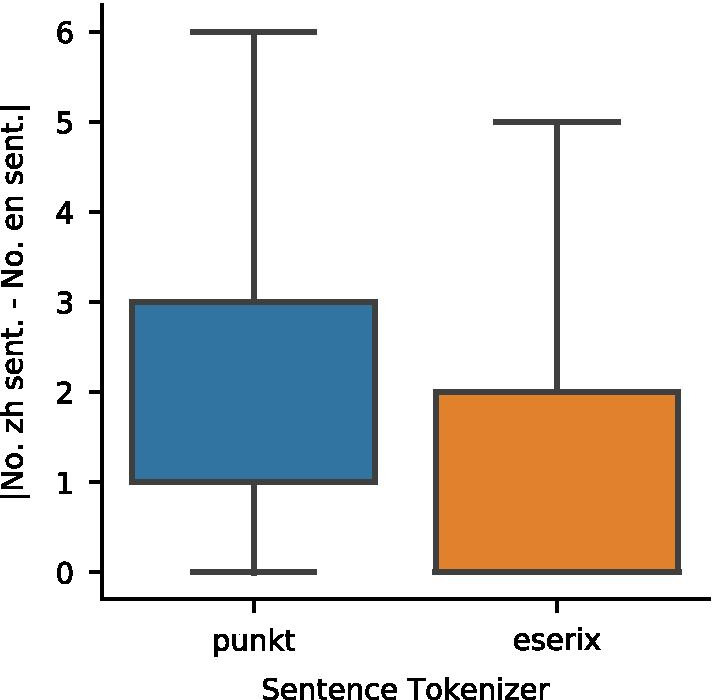
The difference in the number of Chinese and English sentences are smaller in eserix than in punkt. Note that the distributions are left-skewed and the medians overlap with the 1st quartiles

Because no systematic evaluation exists for sentence boundary detection in the biomedical domain, we tested two popular algorithms, punkt and eserix. To compare the two, we plotted the difference in the number of sentences. Because NEJM articles were translated sentence for sentence, the ideal SBD result should have a difference of zero. We found that difference is smaller for eserix (median difference = 0) than punkt (median difference = 1) and thus used it for downstream analysis (Fig. 5).

The two most frequent errors made by punkt were the failure to break at citations (Table 4) and erroneous breaks before open parentheses (Table 5). The latter created difficulty for sentence alignment because the Chinese sentence breaks appear after the close parenthesis. Conversely, eserix did not make these mistakes.

**Table 4:**
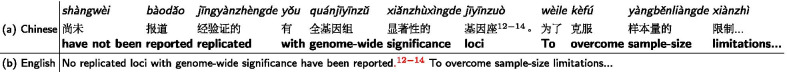
Example of a failure to break two English sentences due to a citation (red text). Corresponding Chinese sentences do not suffer from this problem

**Table 5:**
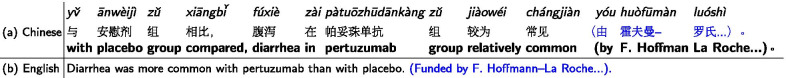
Example of an erroneous break before the blue text. Notice the additional period before the open parenthesis for the English text

### Comparing sentence alignment algorithms

**Fig. 6:**
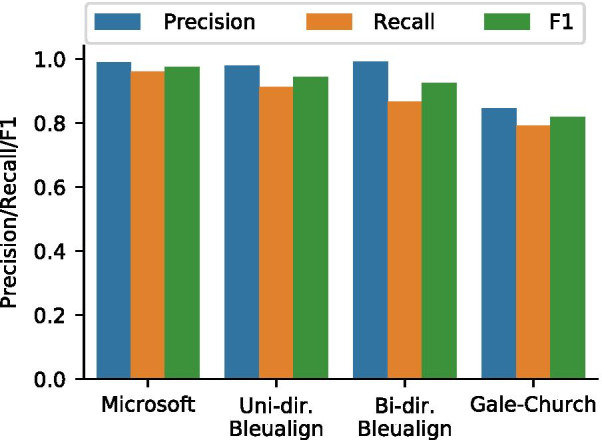
Performance of three sentence aligners on the ParaMed corpus. Uni-directional Bleualign uses translations in zh$\rightarrow$en only, whereas bi-directional Bleualign uses translations in both directions, giving it higher precision but lower recall

To find correspondence between English and Chinese sentences, we tested three types of aligners, Gale-Church (length-based), Microsoft Aligner (lexicon-based), and Bleualign (translation-based), using a manually annotated set of 1,019 sentence pairs (“Sentence alignment” section). It should be noted that Bleualign was tested in both unidirectional (zh$\rightarrow$en) and bidirectional (zh$\leftrightarrow$en) modes. The unidirectional mode has higher recall but lower precision, whereas the bidirectional mode has lower recall but higher precision. The majority of sentence pairs are one-to-one aligned (Table 2) and the performance of all algorithms degrade significantly for one-to-many and many-to-many alignments. Therefore, we focused on one-to-one alignments for this study. The precision, recall, and F1 scores are shown in Fig. 6. The Microsoft Aligner achieved the best F1 score and was used for downstream analysis.

### Statistics of the ParaMed corpus

After the aligned sentences cleaned with bifixer [39], the final corpus contains 1,966 article pairs with a total of 97,441 sentences. We tokenized English sentences with moses [29] and Chinese sentences with Jieba. The English corpus contains 3,028,434 tokens and 55,673 unique tokens. The Chinese corpus contains 2,916,779 tokens and 46,700 unique tokens. All statistics are reported in Table 6.

**Table 6:** Statistics of the ParaMed corpus

Language	Articles	Sentences	Avg. Len.	Tokens	Unique Tokens
English	1,966	97,441	31.08	3,028,434	55,673
Chinese			29.93	2,916,779	46,700

### Machine translation performance

To measure the effect that the ParaMed corpus has on medical translation, we compared the baseline transformer model trained on the WMT18 English-Chinese dataset and a fine-tuned model with the ParaMed corpus (“Hardware and training procedure” section). Although translations are evaluated bidirectionally, it should be emphasized that the ParaMed corpus is translated by human translators from English to Chinese and this bias will influence the machine translation quality [40].

**Fig. 7:**
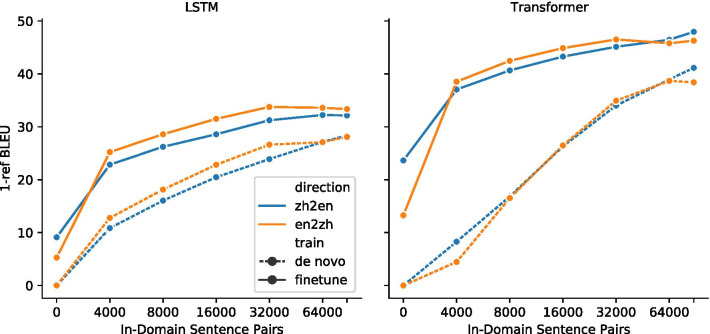
Translation quality improves as the size of the ParaMed corpus increases. The solid lines correspond to the fine-tuned model, whereas the dashed lines correspond to the *de novo* model. The BLUE score for solid line at x = 0 shows the baseline model performance without fine-tuning

To understand the translation quality as a function of in-domain dataset size, we fine-tuned the transformer model on 4,000, 8,000, 16,000, 32,000, 64,000 and all 93,303 sentence pairs (Fig. 7). For both zh$\rightarrow$en and en$\rightarrow$zh models, we saw improvement as the number of in-domain sentence pairs increased. The most significant improvement occurred at 4,000 sentence pairs (en$\rightarrow$zh: +25.3 BLEU; zh$\rightarrow$en: +13.4 BLEU). Translation quality continued to improve as the size of the dataset grows, albeit at a slower pace. Compared with baseline, the full dataset with 93,303 sentence pairs increased the BLEU score by 33.0 (24.3) points in en$\rightarrow$zh (zh$\rightarrow$en) directions. We observed similar effects on the LSTM model. The most significant improvement occurred at 4,000 sentence pairs (en$\rightarrow$zh: +19.9 BLEU; zh$\rightarrow$en: +13.7 BLEU). Compared with the baseline, the full ParaMed dataset increased the BLEU score by 28.1 (23.0) in en$\rightarrow$zh (zh$\rightarrow$en) directions.

To determine whether the pre-training on WMT18 data is necessary, we trained a *de novo* model using only ParaMed data, which was significantly faster than training a baseline model followed by fine-tuning. Compared with *de novo* training with a transformer model, pre-training on WMT18 baseline plus fine-tuning provided a meaningful boost in translation quality. Such boosts were most evident on small in-domain datasets. With 4,000 sentence pairs, pre-training improved the BLEU score by 34.1 (28.8) points for en$\rightarrow$zh (zh$\rightarrow$en) directions. The difference decreased as in-domain dataset grew, dropping to 7.9 (6.8) BLEU points for en$\rightarrow$zh (zh$\rightarrow$en) at the full-set level. A larger in-domain dataset is needed to completely compensate for the gap in translation quality. We observed similar effects for the LSTM model. The fine-tuned model consistently outperformed the *de novo* model for the size of the ParaMed dataset. The gap will likely become smaller as the dataset grows.

### Machine translation error analysis

We showed two examples in this section to illustrate common mistakes made by our models. In the zh$\rightarrow$en direction, the phrase “  ” was correctly translated by the fine-tuned model to “platinum-taxane”, and mistranslated by the baseline model to “Pt-Pseudophyllus” Table 7). The baseline model has not seen the phrase “  ” during training and thus resulted in incorrect decoding. A similar situation occurred at the phrase “  ”. The fine-tuned model was able to correctly translate the phrase into bevacizumab, a chemotherapy medication, whereas the baseline model incorrectly decoded the phrase as “Bavaris mono-repellent”.

**Table 7:**
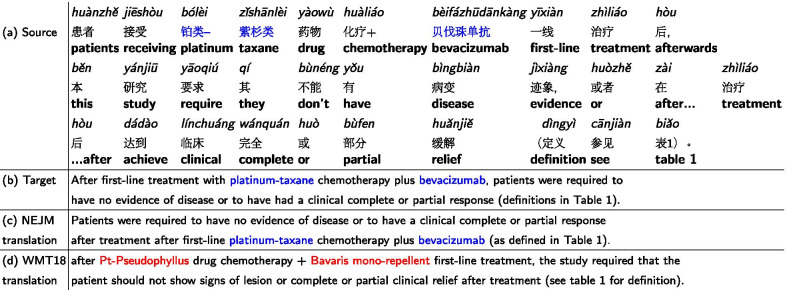
and  were never seen by the baseline model and were translated incorrectly (red text)

**Table 8:**
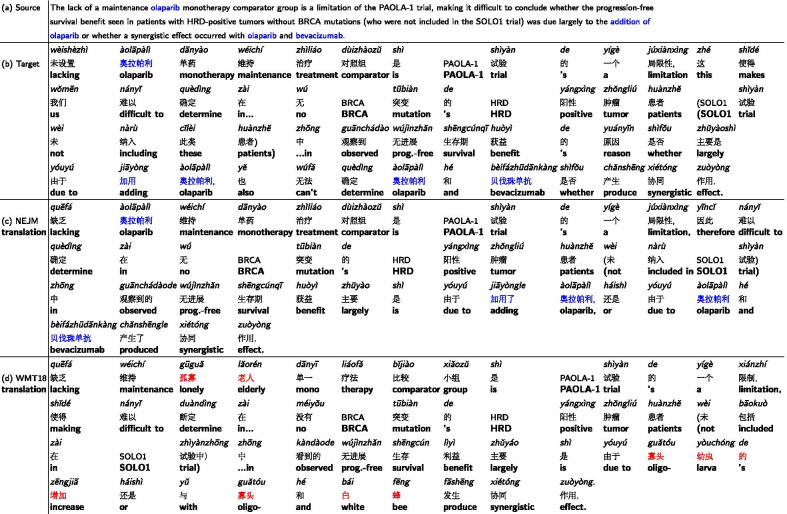
Olaparib and bevacizumab were not seen by the baseline model and were translated incorrectly (red text)

Similar situations occurred for the en$\rightarrow$zh direction (Table 8). Two medications, “olaparib” and “bevacizumab”, were correctly translated by the fine-tuned model as “  ” and “  ”, but incorrectly translated by the baseline model as “  ” and “  ”. Fine-tuning on in-domain data extended the model vocabulary and made it more accurate to decode medical terminology.

## Conclusions

The popularity of neural machine translation models has boosted the need for large datasets. Public releases of many large-scale parallel corpora have significantly improved the quality of machine translation.

Machine translation in the biomedical domain has seen increasing attention in recent years [14, 41, 42]. Biomedical literature is rich in terminology for describing various diseases and biological processes. To add to this challenge, biomedical translation mandates a high standard of translation accuracy because the consequence of misinterpretation in medical decisions can be severe. All these challenges call for the curation of large-scale biomedical parallel corpora.

Despite the need for biomedical parallel text, curation of large-scale corpora have been biased towards European language pairs. Biomedical parallel corpora have been made available across several pairs of European languages, including English, German, Spanish, France, Portuguese, and Polish, to name a few. To our knowledge, there is no English-Chinese parallel corpus in the public domain.

We have presented an English-Chinese parallel dataset in the biomedical domain. We have shown that a baseline model trained on out-of-domain data (WMT18) has limited generalizability to the biomedical domain and that as few as 4000 sentence pairs from the ParaMed dataset substantially improved translation quality. The translation quality continued to improve as the dataset grew. Further, pre-training with the out-of-domain data benefited translation quality, even at the full-set level.

We plan to expand our parallel corpus as New England Journal of Medicine continues to translate more articles. In the future, we would like to include bilingual PubMed abstracts as part of our parallel corpus.

## Data Availability

The code and data are available at https://github.com/boxiangliu/ParaMed.

## Abbreviations

UMLS: Unified Medical Language System
WMT: Conference on Machine Translation
NEJM: The New England Journal of Medicine
SBD: Sentence boundary detection
